# Maternal cannibalism in two populations of wild chimpanzees

**DOI:** 10.1007/s10329-019-00765-6

**Published:** 2019-10-05

**Authors:** Pawel Fedurek, Patrick Tkaczynski, Caroline Asiimwe, Catherine Hobaiter, Liran Samuni, Adriana E. Lowe, Appolinaire Gnahe Dijrian, Klaus Zuberbühler, Roman M. Wittig, Catherine Crockford

**Affiliations:** 1 Department of Primatology, Max Planck Institute for Evolutionary Anthropology, Leipzig, Germany; 2 Budongo Conservation Field Station, Masindi, Uganda; 3 Division of Psychology, Faculty of Natural Sciences, University of Stirling, Stirling, Scotland FK9 4LA UK; 4 Department of Comparative Cognition, Institute of Biology, University of Neuchâtel, Neuchâtel, Switzerland; 5 School of Psychology and Neuroscience, University of St Andrews, St Andrews, UK; 6 School of Anthropology and Conservation, University of Kent, Canterbury, UK; 7 Taï Chimpanzee Project, CSRS, Abidjan, Ivory Coast

**Keywords:** Cannibalism, Chimpanzee, Maternal cannibalism, Parental investment

## Abstract

Maternal cannibalism has been reported in several animal taxa, prompting speculations that the behavior may be part of an evolved strategy. In chimpanzees, however, maternal cannibalism has been conspicuously absent, despite high levels of infant mortality and reports of non-maternal cannibalism. The typical response of chimpanzee mothers is to abandon their deceased infant, sometimes after prolonged periods of carrying and grooming the corpse. Here, we report two anomalous observations of maternal cannibalism in communities of wild chimpanzees in Uganda and Ivory Coast and discuss the evolutionary implications. Both infants likely died under different circumstances; one apparently as a result of premature birth, the other possibly as a result of infanticide. In both cases, the mothers consumed parts of the corpse and participated in meat sharing with other group members. Neither female presented any apparent signs of ill health before or after the events. We concluded that, in both cases, cannibalizing the infant was unlikely due to health-related issues by the mothers. We discuss these observations against a background of chimpanzee mothers consistently refraining from maternal cannibalism, despite ample opportunities and nutritional advantages. We conclude that maternal cannibalism is extremely rare in this primate, likely due to early and strong mother–offspring bond formation, which may have been profoundly disrupted in the current cases.

**Electronic supplementary material:**

The online version of this article (10.1007/s10329-019-00765-6) contains supplementary material, which is available to authorized users.

## Introduction

Filial cannibalism—a form of cannibalism in which a parent consumes its own offspring—is relatively common in some animal taxa (Polis 1981). In fish and reptile species with parental care, for example, eggs can be cannibalized when the costs of investing in the current clutch outweigh the benefits of future reproduction (Klug et al. 2006; Lourdais et al. 2005). Filial cannibalism also exists in mammals (Polis 1981). For example, rodent mothers that give birth to large litters are known to cannibalize some of the infants, although they commonly select ill or handicapped infants, suggesting that this is part of an evolved strategy (DeSantis and Schmaltz 1984).

In primates, incidences of filial cannibalism, in particular by mothers (maternal cannibalism), are extremely rare, and it has been argued that this is due to the high costs of maternal gestation and, at the proximate level, strong mother–infant bonds (Hrdy 2000). If filial cannibalism occurs in primates, it is usually of already-deceased infants, which has been reported in baboons (Altmann 2001), thick-tailed bushbabies (Tartabini 1991), mustached tamarins (Culot et al. 2011), Japanese macaques (Watson et al. 2015), Tonkean macaques (De Marco et al. 2018), and rhesus macaques (Tian et al. 2016). In great apes, maternal cannibalism has been reported in Sumatran orangutans (two cases in a captive population; Dellatore et al. 2009) and bonobos (three instances in two different populations; Fowler and Hohmann 2010; Tokuyama et al. 2017).

For chimpanzees, we are not aware of any reports of maternal cannibalism, despite decades of field research and strong scientific interest in intra-specific violence (Wilson et al. 2014). Moreover, infant mortality is extremely high in chimpanzees with an estimated 20% of infants dying within the first 12 months (Hill et al. 2001). This, combined with the fact that cannibalism in general is not unusual in chimpanzees (Bygott 1972; Pruetz et al. 2017), suggests that chimpanzee mothers refrain from filial cannibalism, despite extensive opportunities.

Here, we provide detailed behavioral accounts of two cases of consumption of newborn infants by their mothers in two chimpanzee subspecies: the Waibira community of Budongo Forest, Uganda (*Pan troglodytes schweinfurthii*), and the East community in the Taï National Park, Ivory Coast (*P. t. verus*). These observations are relevant to current theories of mother–offspring bonds in our closest living relatives, as discussed below.

## Materials and methods

### Study site and subjects

Observations were made in the Waibira and Taï East communities. Habituation of the Waibira community started in 2011 and, by the time of the event, the group comprised 95 named individuals (including 26 males and 31 females older than 12 years) with the majority of individuals habituated to human presence (Samuni et al. 2014). In the Taï East community, behavioral data had been collected since 2007 (Wittig 2018) and the group comprised 36 individuals (including five males and 12 females older than 12 years), all of which were habituated to human presence at the time of the event.

Key individuals involved in the reported event in Waibira:

Monika (MON): Cannibalizing mother, who immigrated to the Waibira community from the neighboring Sonso community in September 2014 (born: July 2003)
Unnamed infant: MON’s first infant born 2–3 weeks prior to the incident (first seen 27th August 2017–last seen 12th September 2017)
Ben (BEN): Top-ranking adult male of the Waibira community (estimated age: 25 ± 2 years)
Lahni (LAN): Low-ranking sub-adult male (estimated age: 15 ± 1 year)
Lokuyu (LKU): Juvenile male (estimated age: 7 years ± 6 months)

Key individuals involved in the reported event in Taï East:

Wandy (WAN): Cannibalizing mother (estimated age: 28 years),
Weh (WEH): Juvenile male (age: 5 years), WAN’s second offspring. WEH’s older sister Woloso emigrated from the group in December 2014
Athos (ATH): Top-ranking male of the Taï East community (estimated age: 21 years)

Others present:

Poseidon (POS; adult male; ca. 19 years old); Fredy (FRE; adult male, ca. 40 years old); Richelieu (RIC; adult male; ca. 13 years old), Indira (IND; adult female, ca. 25 years old), Fatima (FAT; adult female, ca. 16 years old), Ivoire (IVO; infant son of IND, 3 years old), Fiesta (FIE; infant son of FAT, 2 years old), Quarantaine (QUA; orphan sub-adult female, 11 years old), Gia (GIA; orphan sub-adult female, 12 years old); Maimouna (MAI; orphan juvenile female; 6 years old) and Beatrice (BEA; orphan juvenile female; 6 years old).

### Behavioral data

The Waibira observation was made by PF and PT on 16 September 2017; the Taï East observation on 30th May 2015 by AGD. Ad libitum behavioral data were collected from individuals handling the corpse and those within 10 m of the corpse. We recorded any interactions, such as begging, touching, and grooming between individuals involved in or in spatial proximity to the infant consumption. We noted the identities of individuals observed in the party of the cannibalistic individuals. “Party” was defined as all individuals present within an occasionally interrupted visual range (Newton–Fisher 1999).

## Results

### Summary of the events

#### Waibira community (*P. t. schweinfurthii*)

**Fig. 1:**
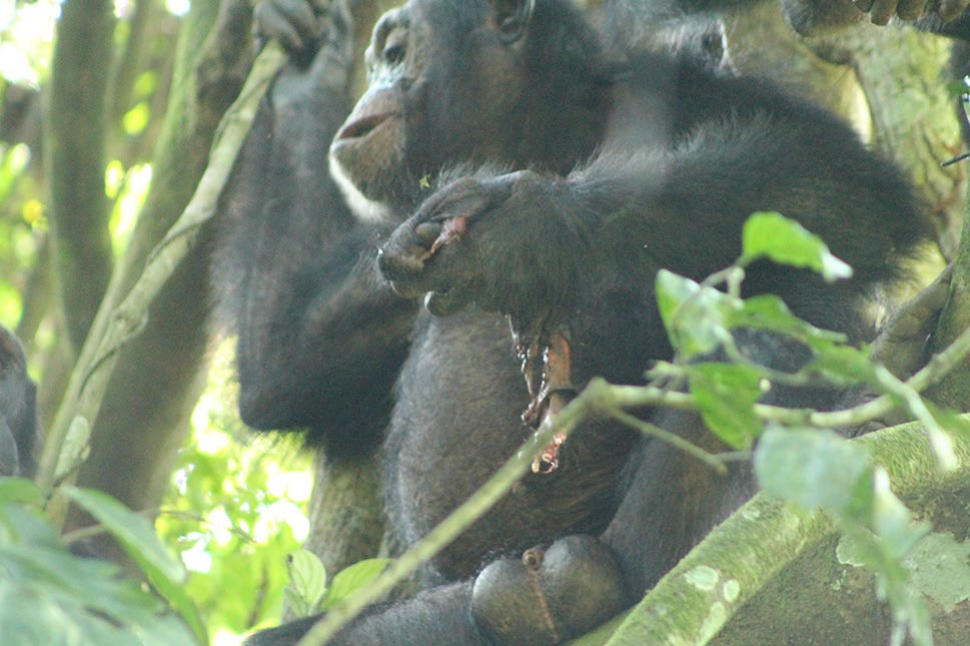
BEN holding and consuming the corpse of MON’s infant

**Fig. 2:**
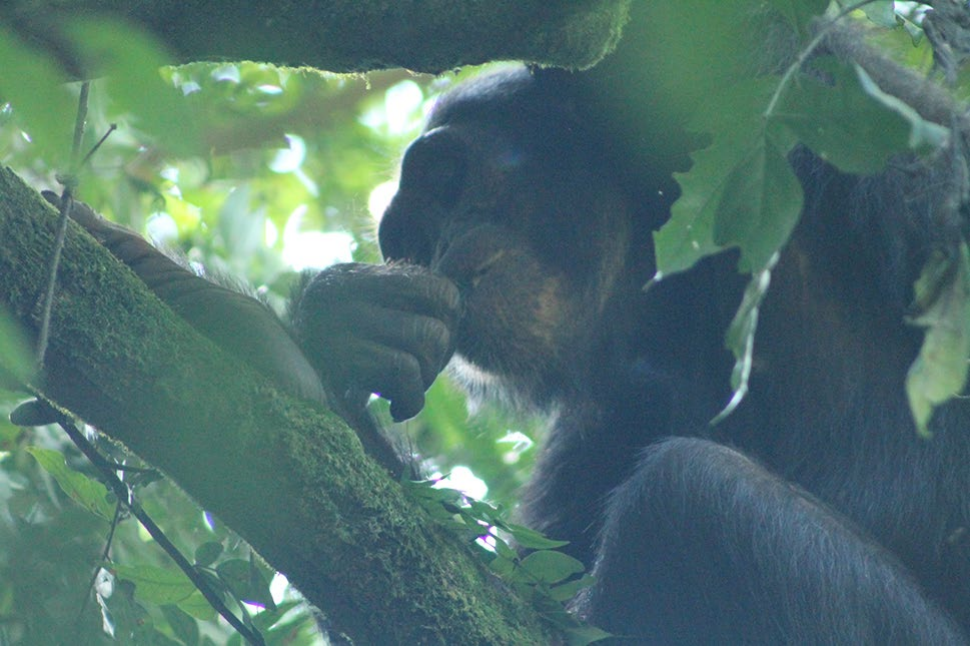
MON consuming her infant

On the 16th of September 2017 at 14:56, LKU was seen carrying the corpse of MON’s infant in his mouth. There were no obvious deformities of the corpse, nor any previous observations of disease in the infant. BEN took the infant corpse from LKU and chased him away, before moving out of the party to begin consuming the corpse while sitting on the ground. After 2 min, BEN was joined by MON and LAN, who observed him feeding. BEN moved into a tree to continue consuming the corpse, supplementing the meat with leaves [Fig. 1; video in Electronic Supplementary Material (Supplementary Material 1)]. MON joined BEN in the tree and groomed him while he ate. MON stopped grooming to begin begging (placing her upturned hand near the mouth of BEN). LAN moved into the tree and began grooming MON. Approximately 30 min after the start of the event, MON begged for and took a piece of the corpse from the hand of BEN and began to feed [Fig. 2; video in Electronic Supplementary Material (Supplementary Material 2)]. LAN stopped grooming MON to beg from BEN (placed his hand near the mouth of BEN) but did not acquire a piece of the corpse. After his failure in begging, LAN pant grunted to BEN and began to groom MON. MON and LAN mutually groomed briefly, before MON returned to grooming and begging from BEN. MON acquired three more pieces of the corpse, which she ate. BEN left the tree with the remainder of the corpse, and was followed by MON and LAN. BEN continued to feed on the ground. LAN left the party. MON resumed begging from BEN and directly fed from the corpse while it was in BEN’s hands. At 16:24, approximately 90 min from the start of the incident, the observers lost both MON and BEN as they moved away into dense vegetation with BEN still carrying the remainder of the corpse.

As the event occurred in the center of the community’s range, it is unlikely that the cannibalized infant belonged to another community. During the month prior to the event, there were no other recorded births, and the only other females that were observed exhibiting signs of late-stage pregnancy all gave birth over the following 2 months. There were no other missing infants during this time. Apart from MON, the only adult female present in the party during the event was LIR, who had a young unweaned infant (LAM, born October 2015), and so could not be the mother of the cannibalized infant. Therefore, it is unlikely that the cannibalized infant belonged to a female other than MON. A detailed chronological description is provided in Electronic Supplementary Material (Supplementary Material 3), as well as videos of the event.

#### Taï community (*P. t. verus*)

On the 27th of May 2015, WAN was first observed with a new infant, after having been absent for 1 week. The infant appeared very small and almost hairless, suggesting a premature birth. On 29th May 2015, the infant was not moving but the observer was unable to confirm it had died. The infant was still carried by WAN the entire day. At 08:46 on 30th May 2015, the infant was declared dead by the observer based on a sustained period of non-movement and not breathing. At 10:30, WAN began to consume the dead infant, sharing some of the meat with her juvenile son, WEH. RIC (an adult male), two other females (IND and FAT) with their infants (IVO and FIE), and two orphans (MAI and BEA) all observed close by as WAN ate the infant, but none actively begged or attempted to take meat from WAN. Shortly after WAN began to consume the infant, the top-ranked male (ATH) joined the party and displayed at WAN, displacing her. At this point, she dropped the dead baby. Her son, WEH, then picked up the infant corpse to inspect it for some moments before WAN returned to retrieve it from WEH. At 11:15, WAN and WEH then left the party; WAN carried the remaining corpse of the infant between her thigh and stomach. WAN and WEH foraged and rested for several hours with WAN carrying the remaining infant corpse throughout and grooming WEH twice during this period. Between 14:25 and 15:50, WAN and WEH joined a party of seven other individuals (BEA, EOL, FRE, GIA, MAI, POS, and QUA), but none were reported to interact with WAN or WEH, nor show an interest in the infant remains. At 15:50, WAN and WEH fissioned from the party and foraged alone once again. At 17:28, the infant corpse was noted to be rapidly decomposing, with many flies swarming on it. At this time, WAN and WEH climbed into a *Dialium aubrevillei* tree to feed. Shortly afterwards, at 17:38, WAN began to make a nest for the night. The next day WAN was observed leaving the nest without the corpse of the infant, which was not seen again by observers.

### Health

Both the Waibira and Taï East communities are regularly monitored by veterinarians for signs of ill health, such as, for example, fatigue, injury, and coughs. Neither MON nor WAN were reported to have any health problems during the reported period.

## Discussion

Mother–infant relationships in mammals, and especially in primates, represent some of the strongest affiliative bonds in the animal kingdom (Broad et al. 2006; Fedurek 2017). Indeed, primate maternal attachment can extend beyond an infant’s death to the extent that primate mothers have been observed to carry and groom the corpse of their infant for weeks or months after death (Biro et al. 2010; Campbell et al. 2016; Fashing et al. 2011; Matsuzawa 1997; Sugiyama et al. 2009; Watson et al. 2015). Cannibalizing their own infants is, therefore, not something that would be expected from primate mothers, despite the nutritional gains and the apparent adaptive value of filial cannibalism observed in species with weak or absent mother–infant bonds (DeSantis and Schmaltz 1984; Polis 1981).

Infant mortality rates in chimpanzees are extraordinarily high for apes (Hill et al. 2001; van Noordwijk et al. 2018), with first-year infant mortality estimated at 20% across populations (Hill et al. 2001). This rate suggests that many chimpanzee mothers experience loss of infants, perhaps even multiple times within their reproductive lifespans. As a result, most females will have an opportunity to cannibalize their deceased offspring. Yet, to our knowledge, such events have not been reported in the scientific literature, despite decades of field studies. Chimpanzee maternal responses to infant deaths are typically characterized by affiliation and carrying of the corpse, not by cannibalization (Biro et al. 2010; Cronin et al. 2011). Indeed, of the 26 direct observations of intra-community infanticide in chimpanzees recorded across several field sites and populations, none was reported to be associated with maternal cannibalism (Lowe et al. 2019; Wilson et al. 2014; Hobaiter pers. comm.).

Our case study of maternal cannibalism in two different communities and sub-species of wild chimpanzees demonstrates that maternal cannibalism, although very rare, is within the behavioral repertoire of chimpanzees, and in line with reports from captive orangutans and wild bonobos (Dellatore et al. 2009; Fowler and Hohmann 2010; Tokuyama et al. 2017). There are several potential explanations for the previous lack of observations in chimpanzees. First, it is possible that maternal cannibalism in chimpanzees is more common. This is a possibility because, in some communities, young mothers can be reclusive after giving birth, actively avoiding contact with other group members, presumably to avoid aggression and infanticide (Nishie and Nakamura 2018; Otali and Gilchrist 2006; Pusey et al. 2008). Indeed, many early infant deaths remain unexplained, with no record of the corpses’ whereabouts, suggesting that maternal furtive behavior may lead to an underestimation of maternal cannibalism rates in wild chimpanzees. However, not all chimpanzee mothers are reclusive immediately following giving birth and, given the considerable amount of field observations of this species across multiple communities, it seems unlikely that chimpanzee maternal cannibalism is a frequent but unseen behavior.

On a proximate level, chimpanzee females may refrain from maternal cannibalism as a consequence of strong mother–offspring attachment and bonding. Indeed, maternal attachment is the predominant explanation for the frequent observations of post-mortem maternal care-giving (such as carrying or grooming) to deceased infants in primates generally (Watson and Matsuzawa 2018), including chimpanzees (Biro et al. 2010; Cronin et al. 2011; Hosaka et al. 2000). In an extreme example, one mother from the Mahale community in Tanzania carried the corpse of her daughter for 4 months (Hosaka et al. 2000). On a proximate level, pregnancy and post-partum hormones stimulate maternal behavior, and the expression of these hormones shifts once females begin reproductive cycling (Hrdy 2000). Therefore, the length of maternal attachment to infant corpses may be regulated by such hormones and, in certain females, the resumption of reproductive cycling may take weeks or months following the loss of an infant leading to prolonged maternal care of corpses. Maternal care-giving involves energetic costs and the post-mortem care-giving observed in primates contrasts with widespread filial cannibalism in fish and some mammal species, sometimes with active killing, for which there are adaptive explanations, namely improving physical condition for future reproductive opportunities (DeSantis and Schmaltz 1984; Lindström 2000; Polis 1981).

If maternal cannibalism is avoided in chimpanzees, which we consider plausible given the available data, we propose several, non-exclusive explanations for why maternal cannibalism can nevertheless occur: (1) environmental stress, (2) peer bonding, and (3) abnormal attachment.

First, it has been suggested that maternal cannibalism is caused by abnormal environments, such as human presence, extreme food deficiency, or unnatural laboratory environments, and therefore represents an abnormal, non-adaptive behavior (Tartabini 1991; Tokuyama et al. 2017). Human presence is unlikely to be the reason for the maternal cannibalism reported in this study as the two mothers were fully habituated to the presence of researchers. Food availability is also unlikely to be a cause of this behavior. In Taï, in one of our study communities, individuals are more likely to access meat when fruit availability is low (Samuni et al. 2018a). Evidence from other communities, however, suggests that overall rates of hunting do not appear to vary with fruit availability (Gilby et al. 2006) or even increase with increased food availability (Gilby and Wrangham 2007; Mitani and Watts 2001). Nevertheless, meat is a valuable source of proteins and minerals, such as sodium, that are poorly represented in the typical fruit-dominated diet of chimpanzees (Boesch and Boesch-Achermann 2000; Reynolds et al. 2015; Tennie et al. 2009). It is thus possible that MON’s or WAN’s behavior could also be related to the nutritional benefits of meat consumption. However, given the large number of documented infant deaths in wild communities that do not lead to maternal cannibalism, or indeed cannibalism of any kind (Lowe et al. 2019; Wilson et al. 2014), we find this an unpersuasive explanation. Future research, however, could examine the abnormal environment hypothesis via non-invasive monitoring of urinary C-peptides as a proxy for nutritional stress (Wessling et al. 2018).

Second, meat consumption in chimpanzees is regularly linked to food sharing, which in turn facilitates the formation and maintenance of social bonds (Wittig et al. 2014). Despite differences in maternal experience (WAN: experienced mother with other offspring in the community; MON: nulliparous, recent immigrant), both mothers took part in the meat sharing with the other community members. Meat sharing is associated with oxytocin secretion, both in Taï (Samuni et al. 2018b) and Budongo (Wittig et al. 2014), potentially acting as a social buffer against stress by down-regulating cortisol secretion [reviewed in McQuaid et al. (2016)]. Sharing the carcass of her own infant, in other words, may temporarily buffer a mother’s stress associated with the death of her infant. It is noteworthy that meat-sharing events involving the mothers of the deceased infants also characterized the three cases of maternal cannibalism in bonobos (Fowler and Hohmann 2010; Tokuyama et al. 2017). However, stress buffering via food sharing is likely a by-product rather than an adaptive strategy. For a chimpanzee mother, the loss of an infant is likely to be extremely stressful, and is costly; relative to most primates and indeed most mammals, reproduction is unusually energetically costly for females of this species (Emery Thompson 2013; Murray et al. 2009). Therefore, if such a stress buffering hypothesis were valid, we would anticipate maternal cannibalism and meat sharing of corpses to be more frequent than current rates of observation.

Finally, on a proximate level, we suggest that the probability of maternal cannibalism increases if maternal attachment is disrupted, possibly when mothers do not perceive the corpse to be their infant or, perhaps concurrently, due to early cessation of the endocrine processes underlying maternal attachment. This seems to be consistent with MON’s behavior, a primiparous mother, who participated in the meat-sharing episode only after another group member was in possession of the partially eaten carcass. The cannibalistic event was not initiated by the mother of the infant but by another individual, making it possible that MON did not consider the meat shared to be her infant. Equally, it may be argued that, because WAN’s infant was small, hairless, and likely premature, it did not fit the perception of an infant chimpanzee.

Despite the fact that infanticide and cannibalism is common in some chimpanzee communities, maternal cannibalism appears to be exceedingly rare. This is likely due to the inhibitory action of strong maternal affiliation, despite potential nutritional benefits. Given apes’ phylogenetic closeness to humans, the responses of non-human ape mothers to the death of their infants is of comparative interest to our own evolution. Throughout human history, cannibalism has been an accepted practice in non-normative and extreme contexts, such as when one’s own survival is at stake (Stoneking 2003), while *filial* cannibalism has been typically shunned across societies (Rosenthal 2018). Our report and others suggest that ape, if not human, females may cannibalize their deceased offspring under some very rare circumstances.

Our case study highlights the importance of reporting rare but important behaviors, such as maternal cannibalism, rather than dismissing them as anecdotal or abnormal. From the possible explanations we proposed, extreme nutritional stress remains a possibility, which could be tested by examining mothers who have just lost their infants. The peer-bonding hypothesis predicts lowered cortisol and increased oxytocin levels in cannibalistic compared to non-cannibalistic mothers—a hypothesis that could be also tested by non-invasive measures of hormone levels, such as from urine sample collection (Wittig et al. 2015). Finally, and most probably based on our observations, it is possible that the probability of maternal cannibalism is increased when maternal attachment is disrupted when the newborn infant has physical abnormalities or if an already-abandoned corpse has been disfigured by ongoing cannibalism by other group members. Testing these hypotheses is clearly not a trivial but certainly a worthwhile task, considering the importance of understanding the evolutionary path to our own abstract and cognitively complex concept of death (Anderson 2016).

## Electronic supplementary material

Below is the link to the electronic supplementary material.

Supplementary material 1 (MP4 11678 kb)

Supplementary material 2 (MP4 33831 kb)

Supplementary material 3 (DOCX 75 kb)
