# Supplementary material for: Maternal cannibalism in two populations of wild chimpanzees
Source: Primates. 2019 Oct 5;61(2):181–7. doi: 10.1007/s10329-019-00765-6 (PMC7080684; doi:10.1007/s10329-019-00765-6)
Supplement: Supplementary file 3 — Supplementary material 3 (DOCX 75 kb) [file 10329_2019_765_MOESM3_ESM.docx]

**Maternal cannibalism in two populations of wild chimpanzees**

Pawel Fedurek, Patrick Tkaczynski, Caroline Asiimwe, Catherine Hobaiter, Liran Samuni, Adriana E. Lowe, Appolinaire Gnahe Dijrian, Klaus Zuberbühler, Roman M. Wittig, Catherine Crockford

Corresponding author: Pawel Fedurek, University of Stirling, Division of Psychology, FK9 4LA, Stirling, Scotland (UK). Tel: [+44 (0)1786 467844](tel:+441786467844); email: pawel.fedurek@stir.ac.uk

**Supplementary Material 3**

Detailed chronological description of the event (16^th^ September 2017)

14:56:00 – BEN was seated on the ground with no individuals within a visible distance.

14:57:11 – BEN ran towards screams heard around 40m away.

14:58:17 – BEN chased LOK, who held MON carcass in the mouth. BEN took away the carcass from LOK. Other individuals were seen in the party: two adult males, three subadult males and one adult female (LOK’s mother).

14:59:14 – BEN traveled for around 30m and started consuming the carcass sitting on the ground.

15:00:01 – MON approached BEN and laid down 0.5 m away from him. She watched BEN consuming the carcass, LAN watched BEN from around 10m. No other individuals were present within a visible distance.

15:05:43 – BEN climbed a tree and rested around 7m from the ground. He resumed consuming the carcass, occasionally grabbing and consuming tree leaves (Figure 1).

15:06:02 – MON climbed the tree, approached BEN at a distance of 1m, and kept watching him.

15:06:18 – LAN climbed the same tree and sit within 3m from BEN.

15:09:36 – MON started grooming BEN.

15:10:06 – MON stopped grooming BEN.

15:10:38 – MON extended her hand and touched BEN’s mouth. Ben kept feeding on the carcass.

15:22:35 - LAN approached MON and started grooming her.

15:26:49 – MON touched BEN’s mouth and took a piece of the carcass. MON started chewing the carcass grabbing and putting it into her mouth (Figure 2).

15:17:11 – LEN stopped grooming MON, extended his hand towards BEN’s mouth but did not succeed in obtaining meat from him.

15:17:43 – LAN started grooming MON, and then MON started grooming BEN.

15:18:31 – LAN stopped grooming MON and pant grunted towards BEN.

15:19:28 – LAN started grooming MON and the two groomed each other mutually.

15:20:40 – LAN and MON stopped grooming.

15:21:07 – MON started grooming BEN.

15:23:24 – MON stopped grooming BEN.

15:20:11 – MON extended hand towards BEN’s mouth (BEN still fed on the carcass).

15:31:05 – MON extended hand towards BEN’s mouth and acquired a piece of carcass from him. She started consuming it.

15:35:04 – MON extended hand towards BEN’s mouth.

15:42:06 – MON extended hand towards BEN’s mouth and acquired a piece of carcass from him.

15:51:13 – MON extended hand towards BEN’s mouth and acquired a piece of carcass. LOK approached BEN, MON and LAN at a distance of 10m and watched them.

15:54:25 – MON extended hand towards BEN’s mouth.

15:57:37 – MON extended hand towards BEN’s mouth.

15:59:08 – MON extended hand towards BEN’s mouth.

16:03:15 – MON extended hand towards BEN’s mouth and acquired a piece of carcass.

16:03:39 – LAN extended his hand towards BEN’s mouth but did not succeed in obtaining meat from him. LOK was no longer seen within a visible distance.

16:03:58 – LAN started grooming BEN.

16:06:24 – MON extended hand towards BEN’s mouth and acquired a piece of carcass.

16:07:06 – LAN stopped grooming BEN.

16:08:13 – LAN extended his hand towards MON’s mouth but did not succeed in obtaining meat.

16:10:55 – MON extended hand towards BEN’s mouth.

16:13:07 – BEN climbed down to the ground and started travelling, MON and LAN followed him immediately.

16:13:10 – LAN pant grunted towards BEN.

16:15:14 – BEN stopped travelling and resumed feeding on the carcass.

16:15:20 – MON approached BEN, extended hand towards his mouth and acquired a piece of carcass. LN was no longer visible.

16:15:56 – BEN started travelling, MON followed him.

16:18:16 – BEN stopped travelling and resumed feeding on the carcass. MON approached BEN, extended hand towards his mouth and acquired a piece of carcass.

16:18:30 – MON bit and ate the carcass directly from BEN’s hands.

16:21:30 – BEN resumed travelling, MON followed him.

16:24:59 – The observers lost contact with MON and BEN.
